# Newborns of Mothers with Venous Disease during Pregnancy Show Increased Levels of Lipid Peroxidation and Markers of Oxidative Stress and Hypoxia in the Umbilical Cord

**DOI:** 10.3390/antiox10060980

**Published:** 2021-06-18

**Authors:** Miguel A. Ortega, Lara Sánchez-Trujillo, Coral Bravo, Oscar Fraile-Martinez, Cielo García-Montero, Miguel A. Saez, Miguel A. Alvarez-Mon, Felipe Sainz, Melchor Alvarez-Mon, Julia Bujan, Juan A. De Leon-Luis, Natalio García-Honduvilla

**Affiliations:** 1 Department of Medicine and Medical Specialties, Faculty of Medicine and Health Sciences, University of Alcalá, Alcalá de Henares, 28801 Madrid, Spain; miguel.angel.ortega92@gmail.com (M.A.O.); oscarfra.7@hotmail.com (O.F.-M.); cielo.gmontero@gmail.com (C.G.-M.); msaega1@oc.mde.es (M.A.S.); maalvarezdemon@icloud.com (M.A.A.-M.); mademons@gmail.com (M.A.-M.); mjulia.bujan@uah.es (J.B.); natalio.garcia@uah.es (N.G.-H.); 2 Ramón y Cajal Institute of Healthcare Research, 28034 Madrid, Spain; 3 Cancer Registry and Pathology Department, Hospital Universitario Principe de Asturias, 28806 Alcalá de Henares, Spain; 4 Service of Pediatric, Hospital Universitario Principe de Asturias, 28801 Alcalá de Henares, Spain; larasancheztrujillo@gmail.com; 5 Department of Public and Maternal and Child Health, School of Medicine, Complutense University of Madrid, 28040 Madrid, Spain; jaleon@ucm.es; 6 Department of Obstetrics and Gynecology, University Hospital Gregorio Marañón, 28009 Madrid, Spain; 7 Health Research Institute Gregorio Marañón, 28009 Madrid, Spain; 8 Pathological Anatomy Service, Central University Hospital of Defence—UAH Madrid, 28047 Madrid, Spain; 9 Department of Surgery, Medical and Social Sciences, Faculty of Medicine and Health Sciences, University of Alcalá, Alcalá de Henares, 28801 Madrid, Spain; sainz.felipe@gmail.com; 10 Angiology and Vascular Surgery Service, Central University Hospital of Defence—University of Alcalá, 28047 Madrid, Spain; 11 Immune System Diseases—Rheumatology and Internal Medicine Service, Center for Biomedical Research Network for Liver and Digestive Diseases (CIBEREHD), University Hospital Príncipe de Asturias, Alcalá de Henares, 28801 Madrid, Spain

**Keywords:** chronic venous disease, pregnancy, oxidative stress, expression of NOX and NOS, HIF-1α, lipid peroxidation

## Abstract

Chronic venous disease (CVD) encompasses a set of disorders of the venous system that have a high prevalence in Western societies and are associated with significant sociohealth costs. Pregnancy is a period in which different hormonal and haemodynamic changes occur that lead to significant changes in the cardiovascular system, increasing the risk of developing venous problems, especially during the third trimester of gestation. In turn, CVD involves a series of local and systemic alterations that can have negative repercussions in pregnancy. In this context, the role of oxidative stress in the pathophysiology of this condition has been shown to significantly affect other vascular structures during pregnancy, such as the placenta. However, the effects of oxidative stress on the umbilical cord in women with CVD have not yet been fully elucidated. Thus, the objective of this study was to analyse the gene and protein expression of the enzymes NOX-1, NOX-2 and iNOS, which are involved in the production of reactive oxygen and nitrogen species, respectively. Similarly, the presence of hypoxia-inducible factor 1-alpha (HIF-1α) in the umbilical cord in women with CVD was compared to that of pregnant control women, and the levels of the lipid peroxidation marker malonyldialdehyde (MDA) in cord tissue and blood was also analysed. Our results support a significant increase in the enzymes NOX-1, NOX-2 and iNOS and HIF-1α and MDA in the umbilical cord tissue and blood of women with CVD. For the first time, our work demonstrates an increase in oxidative stress and cellular damage in the umbilical cords of pregnant women who develop this condition, deepening the understanding of the consequences of CVD during pregnancy.

## 1. Introduction

During pregnancy, a woman’s body undergoes a great variety of changes that significantly affect different organs and systems, mainly the cardiovascular system [1,2,3,4,5]. These adaptations are fundamental for the development of the foetus and the success of the pregnancy. However, they can also be associated with an increased risk of developing various cardiovascular complications [6]. Chronic venous disease (CVD) is a highly prevalent condition in Western society and is characterized by progressive venous hypertension that includes a series of clinical manifestations of varying severity, such as the appearance of varicose veins [7,8,9]. Pregnancy is an important risk factor for the development of CVD, and approximately 1 in 3 pregnant women develop this condition [10,11,12]. During this period, a series of changes in hormonal levels and in the haemodynamics of the blood vessels occur that can lead to an increase in venous pressure, thus contributing to the pathophysiology of CVD [13,14].

CVD has a series of local and systemic consequences that affect the venous wall and other body structures [13]. We have observed that pregnant women with CVD present an increase in markers of placental damage, including angiogenesis and lymphangiogenesis [15], tissue calcifications [16] and lipid profile changes [17], showing the impact of this condition on important tissues for foetal physiology. However, the effect of CVD on the umbilical cord and the blood plasma of the newborn has not yet been fully clarified.

Among the most important effects associated with CVD is an increase in the production of reactive oxygen species (ROS) observable in the venous wall and plasma of affected patients [18,19]. Excessive ROS production is part of a phenomenon known as oxidative stress, which is associated with the development of a wide variety of diseases [20,21] and has a fundamental role in cardiovascular diseases [22].

In turn, oxidative stress is involved in appropriate placental development but is also a key element in placental pathology [23,24]. Among the main producers of ROS are NOXs (NADPH oxidases). Of the different members of this family, the important role of NOX-1 and NOX-2 in both physiological and non-physiological conditions has been described, and they contribute to the pathogenesis of several hypertensive disorders in pregnant women, such as preeclampsia [25]. Several studies have shown an increase in cellular hypoxia associated with the presence of CVD, which can contribute to increased oxidative stress [12,16].

Our previous studies have shown an increase in oxidative stress markers in different placental structures in pregnant women with CVD [26]. Oxidative stress can have important consequences during pregnancy, and several studies have observed significant alterations in the umbilical cord and cord blood of patients with different pathological conditions [27,28,29]. Awareness of these oxidative stress markers could improve our understanding of their environment and their implications for possible neonatal and perinatal pathologies [29]. Additionally, some studies suggest there is a prognostic value to these elevated oxidative stress markers, relating them to ultrasound and cardiotocographic alterations at the time of delivery [30,31]. In the present work, gene and protein expression was studied using real-time PCR (RT-qPCR) and immunohistochemistry of NOX-1, NOX-2 and iNOS and the presence of hypoxia markers (HIF-1α) in the umbilical cords of newborns of pregnant women with CVD. The levels of lipid peroxidation (MDA) in the umbilical cord blood of these newborns were compared with those of controls without gestational CVD.

## 2. Materials and Methods

### 2.1. Experimental Design

An observational, analytical cohort study was conducted with 114 newborns. A total of 62 umbilical cords were studied from newborns of mothers diagnosed with CVD during pregnancy (CVD) who had a median age of 33 years (22–40) and a median gestational age of 40.5 weeks (39–41.5). At the same time, we studied 52 umbilical cords from newborns of pregnant women without CVD during pregnancy (CS), who had a median age of 34 years (27–41) and median gestational age (weeks) of 41 (39–42).

Pregnant women with CVD were free of CVD before inclusion in the study. Exclusion criteria were defined as women with endocrine diseases, such as diabetes mellitus; high blood pressure (HBP); body mass index (BMI) ≥ 25 kg/m^2^; unhealthy habits; active infectious diseases; autoimmune diseases; venous malformations; kidney, heart or lung failure; preeclampsia and/or haemolysis, elevated liver enzymes and low platelets (HELLP) syndrome; intrauterine growth restriction of known cause; pathological lesions such as placental infarcts, avascular villi, delayed maturation and chronic inflammation affecting chorionic villi, Polycystic Ovary Syndrome (PCOS), eclampsia and preeclampsia, along with the emergence of any of these exclusion criteria at any time before delivery and prior evidence of CVD. In this study, older gravid women did not take acetylsalicylic acid as treatment for the prevention of preeclampsia.

The participants were women who had visited their obstetrician at week 32 of gestation. Once informed consent was signed, a clinical history was obtained, and a general physical examination and laboratory measurements were performed. A lower limb examination using Eco-Doppler (portable M-Turbo Eco-Doppler; SonoSite, Inc., Washington, DC, USA) at 7.5 MHz was performed while the women were in an orthostatic position, and the leg was examined by external rotation with support of the contralateral leg. The study included the greater saphenous axis from the inguinal region to the ankle and femoral veins. A study of the small saphenous vein and popliteal vein was also performed standing, with the back to the examiner and the body weight resting on the examined leg. A distal compression manoeuvre was performed. In this study, Valsalva manoeuvres were performed, which when producing a proximal circulatory stop, will allow exploration of venous insufficiency proximal to the detection point, as well as the identification of leakage points (it evaluates the absence of reflux in the femoral-iliac and saphenous-femoral union). The distal compression and decompression manoeuvre was also performed to assess the direction of the truncal venous flow, although it was not a physiological manoeuvre. Pathological reflux was considered when this was greater than or equal to 0.5 s. The classification of CVD in the participating pregnant women was based on the CEAP (Clinical-Etiological-Anatomical-Pathophysiological) [32] Classification of Chronic Venous Disorders, and all of the participants had CEAP classes ≥ 1 (C1 = 59.67% (*n* = 37), C2 = 33.87% (*n* = 21), C3 = 6.45% (*n* = 4)).

Table 1 describes the clinical and demographic characteristics. We have only observed significant differences in the Apgar score in CVD infants at 1 min and 5 min [Apgar score (1 min) = 7.00 [5.00–10.00] CVD, 9.00 [6.00–10.00] CS, ** *p* = 0.0054; Apgar score (5 min) = 8.00 [5.00–10.00] CVD, 9.00 [8.00–10.00] CS, * *p* = 0.0331].

The participants’ pregnancies were routinely monitored at the Hospital Central de la Defensa Gómez Ulla-UAH (Madrid, Spain), and umbilical cord samples were obtained at the time of delivery.

The study was carried out in accordance with the basic ethical principles of autonomy, beneficence, nonmaleficence and distributive justice, and its development followed the rules of Good Clinical Practice, the principles contained in the most recent Declaration of Helsinki (2013) and the Oviedo Convention (1997). The collected data and information complied with the current legislation on data protection (Organic Law 3/5 December 2018 on the Protection of Personal Data and the Guarantee of Digital Rights and Regulation (EU) 2016/679). The project was approved by the Clinical Research Ethics Committee of the Gómez Ulla Military Hospital (37/17).

### 2.2. Tissue Samples

Umbilical tissue biopsies were obtained once the placenta was delivered and used for immunohistochemical, genetic and molecular studies. The biopsy fragments were introduced into 2 different sterile tubes: One containing Minimum Essential Medium (MEM) with 1% antibiotic/antifungal (both from Thermo Fisher Scientific, Waltham, MA, USA) and another containing RNAlater^®^ solution (Ambion, Austin, TX, USA). In the laboratory, the samples were processed in a Class II Telstar AV 30/70 Müller 220 V 50 MHz laminar air flow cabinet (Telstar SA Group, Terrassa, Spain) to maintain a sterile environment.

The preserved samples were kept in 1 mL of RNAlater^®^ at −80 °C until processing for gene expression analysis. The samples preserved in MEM were reserved for histological and immunohistochemical studies.

Blood samples were obtained from the newborns by umbilical vein puncture after delivery. Plasma was obtained from these blood samples for the MDA study. The total volume of sampled blood was transferred from heparinized tubes to sterile centrifuge tubes, which were centrifuged at 1500× *g* for 15 min. Next, the plasma was collected and transferred to 1.5 mL Eppendorf tubes, where it was kept at −80 °C until it was used for the study.

### 2.3. Genetic and Molecular Studies

The amount of cDNA in each sample was quantified by qPCR of the following genes of interest: NOX1, NOX2, iNOS, eNOS and PARP. The results were normalized using the constitutive expression of the TBT gene (TATA-binding protein). De novo primers or specific primers were designed for all genes of interest (Table 2) using the online applications Primer-BLAST and AutoDimer. RNA was extracted using the guanidine-phenol-chloroform-isothiocyanate method. qPCR was performed in a StepOnePlusTM System (Applied Biosystems-Life Technologies, Waltham, MA, USA) using the relative standard curve method. The reaction was performed as follows: 5 μL of each sample containing cDNA at a 1/20 dilution in nuclease-free water was mixed with 10 μL of iQTM SYBR^®^ Green Supermix (Bio-Rad Laboratories, Hercules, CA, USA), 1 μL of forward primer (6 μM), 1 μL of reverse primer (6 μM) and 3 μL of DNase- and RNase-free water in a MicroAmp^®^ 96-well plate (Applied Biosystems—Life Technologies, Waltham, MA, USA), with a total reaction volume of 20 μL.

### 2.4. Immunohistochemical Studies

The samples that were preserved in MEM were washed/hydrated multiple times with antibiotic-free medium to eliminate blood cells and were cut into fragments that were maintained in different fixatives, including F13 (60% ethanol, 20% methanol, 7% polyethylene glycol and 13% distilled H_2_O). After the samples were fixed for the necessary time in each fixing solution, they were dehydrated according to standardized protocols.

The antigen–antibody reaction was detected by the ABC (avidin-biotin complex) method with peroxidase or alkaline phosphatase as the chromogen according to the following protocol: (1) Washing 3 times with 1× PBS for 5 min each; (2) blocking of nonspecific binding sites with 3% bovine serum albumin (BSA) in PBS for 30 min at room temperature; (3) incubation with the primary antibody (Table 3A) diluted in 3% BSA and PBS overnight at 4 °C; (4) rinsing 3 times with PBS for 5 min each; (5) incubation with the secondary antibody bound to biotin (Table 3B) and diluted in PBS for 1 h and 30 min at room temperature; (6) rinsing 3 times with PBS for 5 min each; (7) incubation with the avidin-peroxidase conjugate ExtrAvidin^®^-Peroxidase (Sigma-Aldrich, St. Louis, MO, USA) for 60 min at room temperature (diluted 1/200 in PBS); (8) rinsing 3 times in PBS for 5 min each; (9) development by incubation with diaminobenzidine chromogenic substrate (DAB Kit, SK-4100) (Vector Laboratories, Burlingame, CA, USA). The chromogenic substrate was prepared immediately before exposure (5 mL of distilled water, 2 drops of buffer, 4 drops of DAB, 2 drops of hydrogen peroxide); this technique stains brown. (10) Rinsing 3 times in distilled water for 5 min each to stop the reaction; (11) staining of the nuclei with Carazzi haematoxylin for 5–15 min to obtain contrast; (12) rinsing in running water for 10 min; and (13) mounting in aqueous medium with plasdone. In all immunohistochemical studies, sections of the same tissue were used as a negative control in which incubation with the primary antibody was replaced by incubation in the blocking solution.

For each of the patients in the established groups, 5 sections and 10 fields per section were examined using the IRS-Score method [33]. The preparations were examined under a Zeiss Axiophot optical microscope (Jena, Carl Zeiss, Germany) equipped with an AxioCam HRc digital camera (Jena, Carl Zeiss, Germany).

### 2.5. Lipid Peroxidation Assay

The colorimetric lipid peroxidation assay kit (ab118970, Abcam, Cambridge, United Kingdom) is a suitable tool for the detection of MDA in a variety of samples. The MDA present in the sample reacts with thiobarbituric acid (TBA) to generate MDA-TBA, which is colorimetrically quantified (OD 532 nm). This assay detects MDA levels from 0.1 mol/well MDA (1 mol/well MDA in colorimetry). This study quantified the levels of MDA in umbilical cord tissue samples and the blood plasma of newborns. To perform the experiment, the following protocol was used: (1–3) MDA lysis buffer, phosphotungstic acid and butylated hydroxytoluene (BHT) solution (100×), ready-to-use; the buffer was stored at −20 °C and brought to room temperature before use; (4) TBA solution: A vial of TBA was reconstituted in 7.5 mL of glacial acetic acid and then transferred to another tube, where the final volume was adjusted to 25 mL with distilled water; the sample was mixed well to dissolve the TBA, sonication was performed in an RT water bath and the sample was stored at 4 °C; and (5) MDA standard (4.17 M), ready-to-use: The solution was stored at −20 °C and brought to room temperature before use.

Tissue samples: A total of 20 mg of umbilical cord tissue was used for the analysis. The protocol used was as follows: (1) The tissue was rinsed in cold PBS; (2) the lysis solution (300 mL of lysis buffer with MDA and 3 µL of BHT (100×)) was prepared; (3) the tissue was homogenized in 303 µL of lysis solution (buffer + BHT) using 10–15 passes with a homogenizer on ice; and (4) centrifugation was performed at 13,000× *g* for 10 min to remove the insoluble material and collect the supernatant.

Plasma samples: Samples of 10 µL of blood plasma were used for the two study periods (32 weeks of gestation and 32 weeks after delivery). The protocol used was as follows: (1) Carefully mix 10 µL of plasma with 500 µL of 42 mM H_2_SO_4_ in a microcentrifuge tube; (2) add 125 µL of phosphotungstic acid solution and mix by vortexing; (3) incubate at room temperature for 5 min; (4) centrifuge at 13,000× *g* for 3 min; (5) collect the precipitate and resuspend on ice with 100 µL of distilled water (with 2 µL of BHT (100×)); and (6) adjust the final volume to 200 µL with distilled water.

For the colorimetric assay, a 0.1-M MDA standard was prepared by diluting 10 µL of 4.17 M standard MDA in 407 µL of distilled water. A 2-mM MDA standard was prepared by diluting 10 µL of 0.1 M standard MDA in 490 µL of distilled water. The MDA standard (2 mM) was used, and dilutions were prepared for the standard curve. MDA-TBA was generated by adding 600 µL of TBA to each vial. The samples were incubated at 95 °C for 60 min. Each sample was cooled to room temperature in an ice bath for 10 min. Then, 200 µL of the TBA/standard mixture and 200 µL of the TBA/sample mixture were combined in a 96-well plate for analysis. For greater sensitivity, 300 µL of n-butanol was added to the MDA-TBA precipitate. If there was no separation, 100 µL of 5 M NaCl was added to the mixture and then vigorously stirred. The layers could then be separated by centrifugation (3 min at 16,000× *g*). The MDA-TBA phase was transferred to a new tube, and the n-butanol was evaporated. MDA-TBA was dissolved in 200 µL of distilled water and then placed in a 96-well plate for analysis. The product was immediately measured in a reader at OD 532 nm for the colorimetric assay. The measurement was performed using a Victor 2 multifunction device (Wallac, Victoria, Australia).

### 2.6. Statistical Analysis

For the statistical analysis, the GraphPad Prism^®^ 6.0 (San Diego, CA, USA) program was used, and the Mann–Whitney U test was applied. If the study variables were not quantitative, we used Pearson’s chi-squared test or Fisher’s exact test, when applicable. The data are expressed as the mean with interquartile range. Significance was set at *p* < 0.05 (*), *p* < 0.01 (**), *p* < 0.001 (***).

## 3. Results

### 3.1. The Umbilical Cords of Newborns of Mothers Who Had CVD during Pregnancy Show an Increase in Tissue Markers of Hypoxia

**Figure 1:**
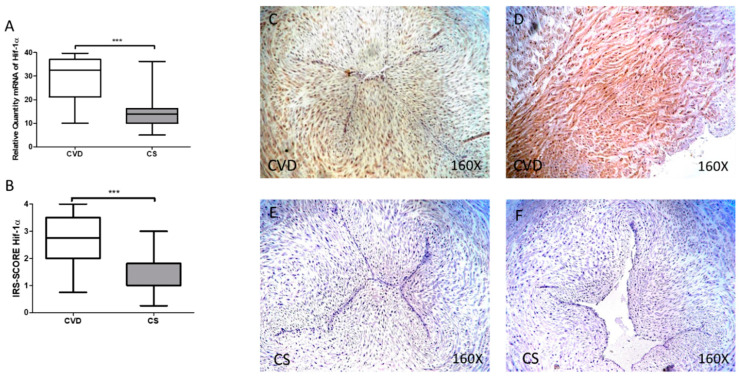
(**A**) mRNA expression levels for Hif-1α by RT-qPCR in the umbilical cord. (**B**) Levels of IRS-SCORE in the umbilical cord. (**C**–**F**) Images showing the immunoexpression of Hif-1α in the umbilical cord. CVD = Newborns of women diagnosed with chronic venous disease during pregnancy. CS = Control without venous pathology. *p* < 0.001 (***).

RT-qPCR and immunohistochemistry revealed the expression of hypoxia markers in the umbilical cords of newborns of women with CVD during pregnancy versus controls without venous pathology (CS). Our results show a significant increase in the gene expression levels of Hif-1α in the umbilical cord of newborns of women who had CVD during pregnancy [CVD = 32.413 (10.051–39.695) UA, CS = 13.944 (5.019–36.189) UA, *** *p* < 0.0001, Figure 1A].

The levels of Hif-1α protein expression, measured by immunohistochemistry, showed a significant increase in the umbilical cords of newborns of women who had CVD during pregnancy [CVD = 2.750 (0.750–4.000), CS = 1.000 (0.250–3.000) UA, *** *p* < 0.0001, Figure 1B]. Localization of Hif-1α at the tissue level was observed at the vein and artery levels and in the extracellular matrix and smooth muscle in the CVD group (Figure 1C,D).

### 3.2. The Umbilical Cords of Newborns Whose Mothers had CVD during Pregnancy Show an Increase in Tissue Markers of Oxidative Stress

**Figure 2:**
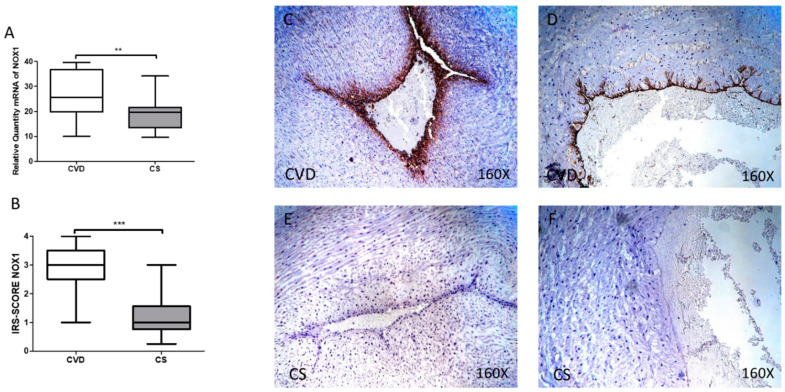
(**A**) mRNA expression levels for NOX1 by RT-qPCR in the umbilical cord. (**B**) Levels of IRS-Score quantification in the umbilical cord. (**C**–**F**) Images showing the immunoexpression of NOX1 in the umbilical cord. CVD = Newborns of women diagnosed with chronic venous disease during pregnancy. CS = Control without venous pathology. *p* < 0.01 (**), *p* < 0.001 (***).

NOX1 gene expression showed a significant increase in the umbilical cord of the newborns whose mothers had CVD during pregnancy [CVD = 26.610 (10.017–39.619) UA, CS = 19.620 (9.631–34.189) UA, ** *p* = 0.0053, Figure 2A]. Immunohistochemistry showed a significant increase in NOX1 protein expression in the umbilical cords of newborns whose mothers had CVD during pregnancy [CVD = 3.000 (1.000–4.000), CS = 1.000 (0.250–3.000) UA, *** *p* < 0.0001, Figure 2B]. Localization of NOX1 at the tissue level was observed at the level of the vein and umbilical artery in the CVD group (Figure 2C,D).

**Figure 3:**
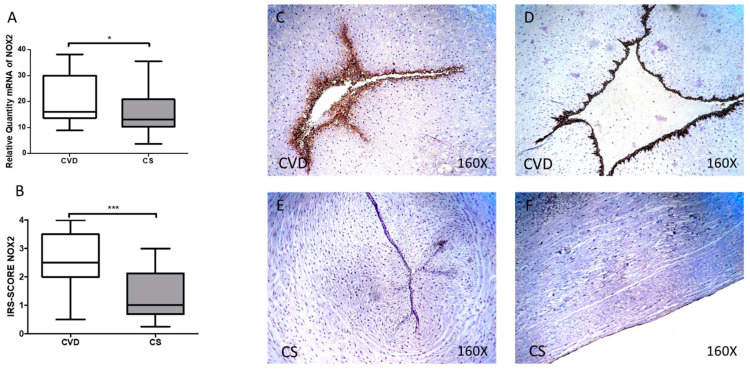
(**A**) mRNA expression levels for NOX2 by RT-qPCR in the umbilical cord. (**B**) Levels of IRS-Score quantification in the umbilical cord. (**C**–**F**) Images showing the immunoexpression of NOX2 in the umbilical cord. CVD = Newborns of women diagnosed with chronic venous disease during pregnancy. CS = Control without venous pathology. *p* < 0.05 (*) and *p* < 0.001 (***).

Similarly, a significant increase in NOX2 gene expression was observed in the umbilical cords of newborns whose mothers had CVD during pregnancy [CVD = 16.090 (8.819–38.198) UA, CS = 13,081 (3.651–35.561) UA, * *p* = 0.00375, Figure 3A]. The analysis of protein expression using immunohistochemical techniques showed that the NOX2 score was significantly higher in the CVD group [CVD = 2500 (0.500–4.000), CS = 1.000 (0.250–3.000) UA, *** *p* < 0.0001, Figure 3B]. Localization of NOX2 at the tissue level was observed at the vein and umbilical artery levels in the CVD group (Figure 3C,D).

**Figure 4:**
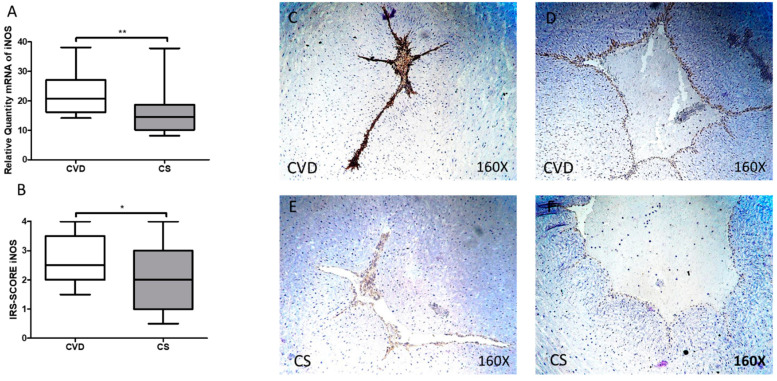
(**A**) mRNA expression levels for iNOS by RT-qPCR in the umbilical cord. (**B**) Levels of IRS-Score quantification in the umbilical cord. (**C**–**F**) Images showing the immunoexpression of iNOS in the umbilical cord. CVD = Newborns of women diagnosed with chronic venous disease during pregnancy. CS = Control without venous pathology, *p* < 0.05 (*) and *p* < 0.01 (**).

RT-qPCR study of iNOS gene expression showed a significant increase in the umbilical cord of newborns whose mothers had CVD during pregnancy [CVD = 20.698 (14.160–38.151) UA, CS = 14.562 (8.189–37.857) UA, ** *p* = 0.0011, Figure 4A]. A significant increase in the iNOS score was observed in the umbilical cord of newborns whose mothers had CVD during pregnancy [CVD = 2.500 (1.500–4.000), CS = 2.000 (0.500–4.000) UA, * *p* = 0.0199, Figure 4B]. The localization of iNOS at the tissue level was also observed at the vein and umbilical artery levels in the CVD group (Figure 4C,D).

### 3.3. Newborns Whose Mothers had CVD during Pregnancy Show Increased Levels of Malondialdehyde at the Tissue and Plasma Levels

**Figure 5:**
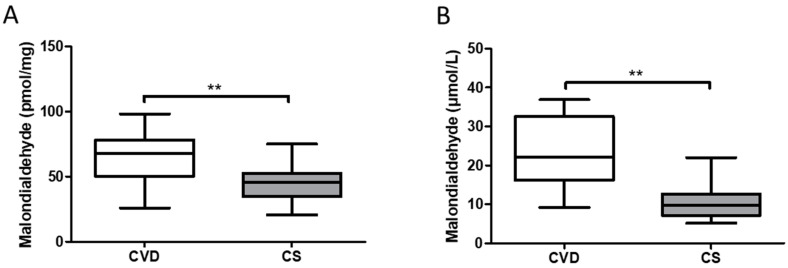
(**A**) Malondialdehyde (MDA) levels in newborn umbilical cord (pmol/mg). (**B**) Malondialdehyde (MDA) levels in newborn plasma (μmol/L). CVD = Chronic Venous Disease, CS = Control without venous pathology. *p* < 0.01 (**).

We observed a significant increase in the levels of malondialdehyde in the umbilical cord tissue of newborns whose mothers had CVD during pregnancy [CVD = 67.683 (26.982–98.197) pmol/mg, CS = 45.662 (20.512–74.891) pmol/mg, ** *p* = 0.0020, Figure 5A]. In parallel, we observed a significant increase in the levels of malondialdehyde in the plasma of newborns whose mothers had CVD during pregnancy [CVD = 22.156 (9.1700–36.916) μmol/L, CS = 9.830 (5.200–21.981) μmol/L, ** *p* = 0.0035, Figure 5B].

## 4. Discussion

In this work, we demonstrated an increase in the gene and protein expression of the enzymes NOX-1, NOX-2 and iNOS as well as HIF-1α in the umbilical cords of pregnant women with CVD. In the same way, we reported a significant increase in the expression of the MDA marker and an increase in its concentration in foetal plasma. Our results are consistent with previous studies that demonstrate greater expression of these components in patients with CVD and in women who develop this condition during pregnancy, which can have important repercussions for certain organs and tissues, such as the placenta [15,19,26].

The gestational process itself is a period of controlled inflammation involving a series of alterations that can lead to low-grade oxidative stress [28,29,31,34]. During pregnancy, there is an increase in the production of ROS, which can have important repercussions for cellular physiology, leading to alterations in the maternal–foetal interface [29,35]. This study shows for the first time how CVD promotes an increase in ROS and markers of oxidative stress in the umbilical cords of pregnant women with this condition. In this way, increased ROS production, orchestrated by a high expression of NOX-1 and NOX-2, could contribute to the alterations detected in this organ. The presence of high levels of NOX and ROS as an indicator of vascular damage has been widely demonstrated in various studies [36,37]. This abnormal increase in NOX-1 and NOX-2 could critically affect the blood vessels of the cord, which in turn can negatively affect its function. NOX-1 is the main producer of ROS during pregnancy, and its levels are substantially increased under pathological conditions [38,39,40]. NOX-2 levels are also elevated due to the development of gestational complications and are closely involved in the deregulation of some key cellular products, such as vascular endothelial growth factor (VEGF), and in the process of angiogenesis [41]. A previous study demonstrated an increase in the expression of VEGF in women with CVD that was part of the pathophysiology of this disease in placental tissue [16]. Therefore, this increase in the expression of NOX-2 could also be related to the alteration of angiogenesis and its key products in the umbilical cords of women with CVD.

However, the mechanisms that lead to the overexpression of NOX-1 and NOX-2 have not yet been completely clarified. Some studies suggest that the expression of these components is induced by the presence of some proinflammatory cytokines, such as TNFα [42].

iNOS is an important inducer of the production of NO and RNS and regulates a wide variety of cellular processes in situations of tissue stress [26]. Our work shows that there is a significant increase in the expression of this component in the umbilical cords of women with CVD. Similarly, previous studies have shown the impact of increased iNOS under pathological conditions such as obesity or preeclampsia [43], in which it induces endoplasmic reticulum stress and apoptosis [44]. The importance of iNOS expression in endothelial cells in the umbilical cord has been demonstrated; it regulates blood flow in this structure [45]. The expression of iNOS is regulated at various levels by the presence of various transcription factors, mainly in a proinflammatory environment. However, some authors argue that iNOS is not produced locally in this tissue but is transported through the maternal blood [46]. A previous study demonstrated an increase in iNOS in pregnant women with CVD, and CVD could explain the increase in the expression of this product in affected women [26]. To this end, our previous studies demonstrated that newborns of mothers with CVD during pregnancy experience significant acidification of the foetal pH measured in the umbilical vein at birth.

Similarly, we also found an increase in the expression of HIF-1α, an element that is frequently deregulated in venous conditions [12,26]. HIF-1α is a master regulator of the cellular response to hypoxia [12]. The role of this element in oxidative stress is certainly controversial. On the one hand, HIF-1α targets multiple genes and mitochondrial products to decrease the production of ROS [47]. On the other hand, some authors have reported an increase in the production of NO in response to cellular hypoxia in some pregnancy complications, such as preeclampsia [47]. Different intermediaries, such as miRNAs or transcription factors, that may be involved in the relationship between HIF-1α and ROS/RNS have been described [48]. Future research will be necessary to delve into the different pathophysiological mechanisms that are altered in the umbilical cords of women with CVD.

Finally, we also observed an increase in MDA in the umbilical cords of women with CVD, which may be an indicator of increased lipid peroxidation in the tissue [18,26]. MDA is a secondary product obtained from the chemical reactions of ROS/RNS with polyunsaturated fatty acids and arachidonic acid derivatives with a high mutagenic capacity [18]. Thus, the increase in MDA could be associated with damage to the cellular lipid components in the umbilical cords of women with CVD, which can also lead to accelerated tissue ageing [26]. In addition, our results show a significant increase in the expression of this component in foetal blood plasma. The usefulness of measuring this component in physiological and pathological conditions during pregnancy has been demonstrated [28,49]. Importantly, the presence of MDA in cord blood could be an indicator that the pro-oxidative status also affects the foetus, which can lead to the development of multiple neonatal pathologies [31,50].

## 5. Conclusions

In this work, we have shown for the first time the existence of a significant increase in the gene and protein expression of NOX-1, NOX-2 and iNOS oxidative stress markers, HIF-1α and the lipid peroxidation marker MDA in the umbilical cords of newborns whose mothers had gestational CVD and in foetal plasma. Future research could aim to evaluate the long-term impact of these events, such as the epigenetic effect, as well as expanding the sample size of the cohort to perform a classification based on the stage and degree of the CVD. Such studies will allow the identification of new targets that can be used in the clinical management of this condition. Understanding the role of oxidative stress in neonatal and perinatal pathology seems to be key to the diagnosis and early management of these entities. At the same time, further studies are required to determine the prognostic value of these markers for foetal and neonatal development and the possibility of developing panels of oxidative markers that are useful in clinical practice. 

## Figures and Tables

**Table 1:** Demographics and clinical characteristics of pregnant women and newborns of women diagnosed with chronic venous disease during pregnancy (CVD) and Control without venous pathology (CS), gr = grams, cm = centimetres, *p* < 0.05 (*) and *p* < 0.01 (**).

	CVD (*n* = 62)	CS (*n* = 52)
Median age [IQR]	33 [22,23,24,25,26,27,28,29,30,31,32,33,34,35,36,37,38,39,40]	34 [27,28,29,30,31,32,33,34,35,36,37,38,39,40,41]
Gestational age (wk) median [IQR]	40.5 [39–41.5]	41 [39,40,41,42]
Previous pregnancies	33 (53.2%)	19 (36.5%)
C-section delivery	12 (19.4%)	9 (17.3%)
CEAP		
C1	37 (59.7%)	0 (0%)
C2	21 (33.8%)	0 (0%)
C3	4 (6.5%)	0 (0%)
Newborn body weigh (gr)	3190.00 [2069.00–3890.00]	3250.00 [4050.00–2055.00]
Newborn size (cm)	48.00 [41.00–50.00]	49.00 [42.00–53.00]
Apgar score (1 min) **	7.00 [5.00–10.00]	9.00 [6.00–10.00]
Apgar score (5 min) *	8.00 [5.00–10.00]	9.00 [8.00–10.00]

**Table 2:** Primer sequences used in RT-qPCR and temperature (Tm).

GENE	SEQUENCE Fwd (5′→3′)	SEQUENCE Rev (5′→3′)	Temp
TBP	TGC ACA GGA GCC AAG AGT GAA	CAC ATC ACA GCT CCC CAC CA	60 °C
Hif-1α	ACGTGTTATCTGTCGCTTTGAG	ATCGTCTGGCTGCTGTAATAATG	59 °C
iNOS	CCT TAC GAG GCG AAG AAG GAC AG	CAG TTT GAG AGA GGA GGC TCC G	61 °C
NOX1	GTT TTA CCG CTC CCA GCA GAA	GGA TGC CAT TCC AGG AGA GAG	55 °C
NOX2	TCC GCA TCG TTG GGG ACT GGA	CCA AAG GGC CCA TCA ACC GCT	60 °C

**Table 3:** Primary (**A**) and secondary (**B**) antibodies used in the immunohistochemical studies performed, showing the dilutions used and the protocol specifications.

(**A**)				
**Antigen**	**Species**	**Dilution**	**Provider**	**Protocol Specifications**
NOX 1	Rabbit	1:250	Abcam (ab78016)	10 mM Sodium citrate pH = 6 before incubation with blocking solution
NOX 2	Goat	1:500	Abcam (ab111175)	100% Triton 0.1% in PBS, 10 min, before incubation with blocking solution
iNOS	Rabbit	1:350	Abcam (ab95866)	10 mM Sodium citrate pH = 6 before incubation with blocking solution
Hif-1α	Mouse	1:800	Abcam (ab16066)	EDTA at pH 9 before incubation with blocking solution
(**B**)				
**Antigen**	**Species**	**Dilution**	**Provider**	**Protocol Specifications**
IgG (Mouse)	Goat	1:300	Sigma-Aldrich (F2012/045K6072)	--------------------
IgG (Rabbit)	Mouse	1:1000	Sigma-Aldrich (RG-96/B5283)	--------------------
IgG (Goat)	Mouse	1:100	Sigma-Aldrich (A5420)	--------------------

## Data Availability

The data used to support the findings of the present study are available from the corresponding author upon request.
